# Interaction Mechanism between OVA and Flavonoids with Different Hydroxyl Groups on B-Ring and Effect on Antioxidant Activity

**DOI:** 10.3390/foods11091302

**Published:** 2022-04-29

**Authors:** Wenna Zhou, Chunyan Peng, Danshu Wang, Jinlin Li, Zongcai Tu, Lu Zhang

**Affiliations:** National R&D Center of Freshwater Fish Processing and Engineering Research Center of Freshwater Fish High-Value Utilization of Jiangxi, College of Life Science, Jiangxi Normal University, Nanchang 330022, China; zhouwenna0622@163.com (W.Z.); pengchunyan2021@163.com (C.P.); 13070674597@163.com (D.W.); lijinlin405@126.com (J.L.); 004756@jxnu.edu.cn (Z.T.)

**Keywords:** ovalbumin, flavonols, intermolecular interaction, antioxidant activity

## Abstract

Ovalbumin (OVA) is a common carrier with high efficiency to deliver flavonoids. The aim of this study was to investigate the interaction mechanism of OVA and four flavonoids (quercetin (Que), myricetin (Myri), isorhamnetin (Ish), and kaempferol (Kaem)) with similar structures by fluorescence spectra, SDS−PAGE, FT−IR, and molecular docking analysis, and the effect on the antioxidant abilities of flavonoids was also evaluated. Results indicated that the antioxidant activity of flavonoids was positively correlated to the number of phenolic hydroxyl groups of on the B-ring, and weakened when the C-3′ position was replaced by a methoxy group. The addition of OVA enhanced the antioxidant activity of Que/Kaem, while it masked the antioxidant activity of Myri. The formation of Que/Myri/Ish/Kaem−OVA complexes was a spontaneous exothermic process driven mainly by hydrogen bond and van der Waals force, which could result in the change in OVA conformation and induce the transformation of α-helix to β-sheet. Among these, Kaem exhibited the strongest binding ability with OVA, and showed the greatest impact on the secondary and conformational structure of OVA, followed by Que. The hydroxylation of C-3′ and methoxylation of C-5′ weaken the interaction of Kaem with OVA. Molecular docking analysis suggested that Que, Myri, Ish, and Kaem formed six, three, five, and four hydrogen bonds with OVA, and the number of hydrogen bonds was not positively correlated with their binding constants. Our findings can provide a theoretical basis for the application of OVA on improving the antioxidant activity of flavonoids, and may help to explain the delivery efficiency of OVA on different bioactive constituents.

## 1. Introduction

**Figure 1:**
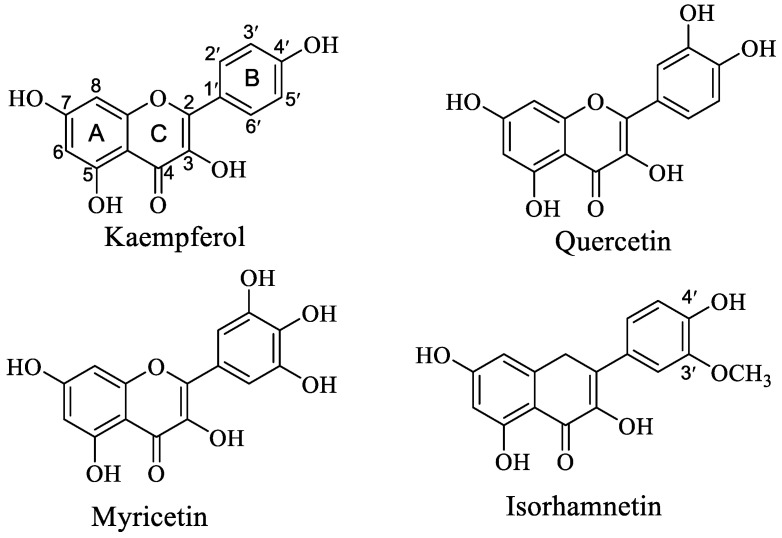
Chemical structure of kaempferol, quercetin, myricetin, and isorhamnetin.

Flavonoids are widely distributed in plants and fruits, and are the most abundant type of naturally occurring polyphenols. Their primary structure consists of two groups: benzopyran (A and C ring) and phenyl (B ring). Based on the connection type of benzopyran with phenyl and the change in C-ring, it can be further divided into flavanones, flavonols, flavones, isoflavones, and anthocyanins [1]. Their bioactivities also varied depending on structural changes, such as the substitution position, type, and number, and even configuration change [2]. Quercetin (Que), myricetin (Myri), kaempferol (Kaem), and isorhamnetin (Ish) are the major flavonoid aglycones present in many fruits, vegetables, herbals, et al. [3]; their structures are different in the number of -OH in the B-ring (Figure 1).

A lot of research indicated that Que/Myri/Kaem/Ish and their derivatives have many physiological activities, such as antioxidant, antibacterial, anti-cardiovascular, antiosteoporosis, antidiabetic, and anticancer effects [4,5]. Currently, many approaches have been applied to improve the bio-accessibility, water solubility, and bioactivities, such as covalent and noncovalent binding with protein, delivery system, and structural modification [6,7,8].

Eggs are one of the most important protein sources in daily diet; they have better nutritional value than many other proteins. As the main protein in eggs, ovalbumin (OVA) is composed of 386 amino acids with molecular weight of 45 kDa; these amino acid residues are entangled and folded to form globular proteins, and most of the secondary structures are α-helix and β-sheet [9]. It was proved that OVA shows excellent foaming ability, emulsification and antioxidant capacities, and has been widely used in food processing to improve the quality and nutrition of products [10]. In addition, OVA is used as a carrier of small molecules to improve its biological activities, stability, or solubility. Gou, et al. [11] found that OVA enhanced the anti-inflammatory activity and bioavailability of epigallocatechin gallate through noncovalent binding. Upon combining with OVA, the crystal structure of curcumin was changed, and the antioxidant activity of OVA−curcumin complexes was higher than that of curcumin alone [12]. Meanwhile, obvious masking effects on the antioxidant properties of polyphenols were also observed on milk and soy protein; this ascribes to the differences in proteins and polyphenols [6]. Until now, a handful of researchers analyzed the influence of polyphenol structure on its interaction with milk protein, but little is known with egg protein.

Our previous research indicated that Que, Myri, Kaem, and Ish could inhibit the glycosylation of OVA by altering its conformation structure, capturing dicarbonyl radicals, and altering the microenvironment surrounding tryptophan; the ability was varied depend on their structures [13]. However, the effect of OVA on the antioxidant activity of Que/Myri/Kaem/Ish and their interaction mechanism is still not clear. The effect of hydroxyl substitutions also needs further exploration. Therefore, in this study, the antioxidant activity of Que/Myri/Kaem/Ish upon binding with OVA was analyzed by ABTS^+^··scavenging ability and Fe^3+^ reducing power. The interaction mechanism between OVA and Que/Myri/Kaem/Ish was studied by fluorescence spectroscopy and molecular docking. Changes in the structure and molecular weight of OVA were studied by circular dichroism (CD), Fourier transform infrared spectroscopy (FT−IR), and sodium dodecyl sulfate polyacrylamide gel electrophoresis (SDS−PAGE). This might provide a better understanding for the interaction of flavonols with protein, and a new idea for improving the bioactivities of flavonols.

## 2. Materials and Methods

### 2.1. Materials

Ovalbumin (from chicken egg white, lyophilized powder, >98%) was purchased from Sigma−Aldrich chemical Co. (St. Louis, MO, USA). Que, Ish, Kaem, Myri, and other reagents were analytical grade and acquired from Solarb chemical company (Shanghai, China).

### 2.2. Sample Preparation

The OVA solution (10 mg/mL) was prepared with 0.2 M PBS buffer (pH 7.4), and flavonols solutions (10 mM) were dissolved in pure methanol. Then, 0.15 mL of Que/Ish/Kaem/Myri was added into 3.0 mL of OVA drop by drop and allowed to shake at room temperature for 2 h. The mixtures were centrifuged, and the supernatants were collected and stored at 4 °C for use.

### 2.3. Intrinsic Fluorescence Analysis

Effects of Que/Myri/Kaem/Ish (6.62 μM) on the intrinsic fluorescence spectra of OVA (1.0 mg/mL) were studied using an F-7000 fluorescence spectrophotometer (Hitachi, Tokyo, Japan) according to a previous report [14]. The emission spectra at 300−400 nm were recorded with the excitation wavelength at 280 nm. The slit width and voltage were 2.5 nm and 700 V, respectively.

### 2.4. FT−IR Spectroscopy

The binding of flavonols with OVA was analyzed by using FT−IR spectrometer (Spectrum One, Perkin Elme, Shelton, CT, USA) equipped with a deuterium triglyceride sulfate crystal (DTGS) detector. The samples prepared according to the method described in Section 2.2 were freeze-dried prior to FT−IR analysis. Spectra were recorded at a range of 7800−350 cm^−1^ for 32 scans with the resolution of 4 cm^−1^. All tests were conducted in triplicate.

### 2.5. SDS−PAGE Analysis

The SDS−PAGE was conducted according to the method of Shen, et al. [15]. Samples were 10-fold diluted and mixed with loading buffer in a ratio of 3:1 (*v*/*v*) and boiled for 5 min. The mixtures were centrifuged at 5000× *g* for 3 min; 10 μL supernatant was loaded on the gel lanes for electrophoresis. The concentrated gel of 5% and the separated gel of 12% were used to separate the proteins. Finally, the gels were stained with 0.05% Coomassie Brilliant Blue G-250 and detained with 50% (*v*/*v*) methanol containing 7.5% (*v*/*v*) acetic acid. Molecular weight marker at 14.4−97.4 kDa was used to estimate the molecular weight change.

### 2.6. Circular Dichroism Spectroscopy Analysis

The effect of Que/Ish/Kaem/Myri on the secondary structure of OVA was analyzed with CD spectrometer (French Bio−Logic SAS, Claix, French) according to Stojadinovic, et al. [16]. The spectra of OVA (0.1 mg/mL) in the presence or absence of flavonols were recorded from 190 nm to 250 nm under constant nitrogen flushing at 37 °C. The spectra of Que/Ish/Kaem/Myri at corresponding concentration were subtracted to eliminate the effect of flavonols. The ellipticity ([θ], degree·cm^2^/dmol) of secondary structures were calculated using the online analysis software DichroWeb (DichroWeb2001, Birkbeck College, London, UK, http://dichroweb.cryst.bbk.ac.uk/html/process.shtml, accessed on 5 August 2021).

### 2.7. Fluorescence Titration Experiment

The intrinsic fluorescence of OVA in the presence of 0.0−12.98 μM of Que/Kaem/Ish/Myri was measured using an F−7000 Fluorescence spectrometer (Hitachi, Tokyo, Japan) at 298, 304, and 310 K, respectively [17]. The protein concentration was fixed at 1.0 mg/mL, and the excitation and emission wavelengths were 280 nm and 300~400 nm, respectively. The scanning voltage and slit width were set at 700 V and 2.5 nm, respectively.

The fluorescence quenching type and quenching constants were analyzed by the Stern−Volmer equation:

(1) $$\frac{F_{0}}{F}=1+K_{SV}\left[Q\right]=1+K_{q}\tau_{0}\left[Q\right]\;$$

where *F* and *F*_0_ are the fluorescence intensity of OVA with and without flavonols, respectively; [*Q*] is the concentration of flavonols; *K_q_* is the fluorescence quenching rate constant; *K_sv_* is the Stern−Volmer quenching constant; and *τ_0_* (10^−8^ s) is the average fluorescence lifetime of OVA without quencher.

For static quenching, the binding constant (*K_a_*) and the number of binding points (*n*) of Que/Myri/Kaem/Ish to OVA can be calculated via the double logarithmic equation:

(2) $$\log\frac{F_{0}-F}{F}=\log\;K_{a}+n\;\log[Q]$$

The thermodynamic parameters enthalpy change (*ΔH*), entropy change (*ΔS*), and free energy change (*ΔG*) during the formation of OVA−Que/Myri/Kaem/Ish complexes were calculated by Van ’t Hoff equation [18]:

(3) $$\ln K_{a}=-\Delta H/RT+\Delta S/R$$

(4) ΔG°=ΔH°−TΔS°

where *R* is the gas constant 8.314 J·mol^−1^·K^−1^; *T* is the reaction temperature (298, 304, 310 K); and *K_a_* is the binding constant of Que/Myri/Kaem/Ish with OVA at corresponding temperature.

### 2.8. Molecular Docking Analysis

AutoDocktools 1.5.6 (Scripps Research, La Jolla, CA, USA) was used to analyze the interaction between OVA and Que/Ish/Kaem/Myri. The 3D structure of OVA (PDB entry: 1 UHG)) was downloaded from Protein Data Bank (https://www.rcsb.org/, accessed on 5 August 2021). The 3D structures of Que, Ish, Kaem, and Myri were drawn with ChemBio3D Ultra 14.0 (PerkinElmer, Waltham, MA, USA) and saved as mol files in the lowest energy. The structures of OVA and Que/Ish/Kaem/Myri were further processed with AutoDockTools 1.5.6, and saved as pdbpt files for further docking. The center co-ordinates of the docking box were (6.52, 21.49, 31.95), the number of grid points in XYZ axis was set as 100 × 100 × 120, and the spacing of grid points is 0.375 Å. Lamarckian GA genetic algorithm was used for docking calculation; default values for other parameters were used. The docking was run 100 times, and results were visualized by Discovery Studio.

### 2.9. Antioxidant Abilities Assays

The antioxidant abilities were measured by ABTS^+^· scavenging ability [19] and Fe^3+^ reducing power assays [20] according to previous reports with minor modifications. For ABTS^+^· scavenging ability, the samples were reacted with ABTS^+^· at a 1:3 volume ratio. The results were expressed as percentage inhibition (%). In terms of Fe^3+^ reducing ability, sample (100 μL) was mixed with 1.0% K_3_[Fe(CN)_6_] solution (250 μL); after 20 min of incubation at 50 °C, the reaction was terminated with 10% TCA. Then, distilled water (0.5 mL) and 0.1% FeCl_3_ (0.2 mL) solution were added, the mixtures were centrifuged at 3000 g for 3 min, and 200 μL of supernatants were pipetted and used for UV (UV−2900, Hitachi, Tokyo, Japan) absorbance measurement at 700 nm. The absorbance was used to reflect the antioxidant activity directly.

### 2.10. Statistical Analyses

All data were analyzed using SPSS Statistics 22.0 (SPSS IBM., Chicago, IL, USA). Significant differences analysis between data was performed using the one-way analysis of variance (ANOVA) test (*p* < 0.05). All experiments were repeated three times, and the results were expressed as mean ± variance (mean ± SD).

## 3. Results and Discussion

### 3.1. Intrinsic Fluorescence Spectra of OVA Affected by Que/Myri/Kaem/Ish

**Figure 2:**
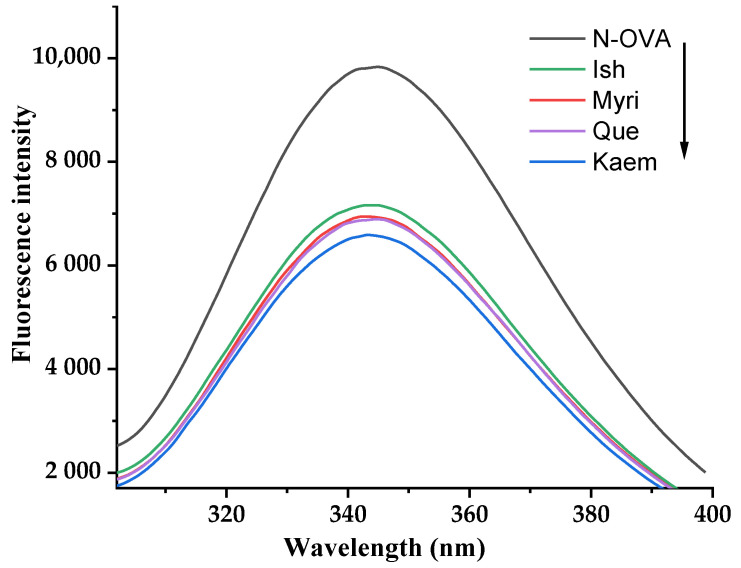
Intrinsic fluorescence spectra of native OVA in the presence of Que, Myri, Kaem, and Ish.

Alteration in the intrinsic fluorescence of protein can reflect its conformation changes. Influences of Que/Myri/Kaem/Ish on the intrinsic fluorescence of OVA are shown in Figure 2; natural OVA has the strongest fluorescence intensity, and, when Que, Myri, Kaem, and Ish were mixed, the values were decreased from 9828 to 6891, 6943, 6586, and 7164, respectively. The corresponding λem were shifted from 344.8 to 344.6, 343, 343.2, and 343.8 nm. The OVA molecule contained 33 inherent fluorescent chromophores, including 3 trytophan (Trp), 10 tyrosine (Tyr), and 20 phenylalanine residues. The decrease in fluorescence intensity and the blue shift of λ_max_ indicated that Que, Myri, Kaem, and Ish could induce the conformation unfold of OVA, motivating chromophores to expose to a more polar microenvironment [21]. Similar experimental results were observed on the interaction between three alkaloids and OVA [22]. Kaem has the strongest fluorescence quenching effect on OVA, followed by Myri and Que; Ish showed the lowest quenching ability. This indicated that Que, Myri, Kaem, and Ish bound to OVA and quenched the intrinsic fluorescence. In addition, the number of hydroxyl groups negatively contributed to the fluorescence quenching ability, and the hydroxylation of C-3′ and C-5′, as well as the methoxylation of C-5′, alleviated the quenching effect on OVA fluorescence; the effect of methoxy group was stronger than that of phenolic hydroxyl group. This trend was consistent with antioxidant activity. Xiao, et al. [23] found the fluorescence quenching effect of dihydroartemisinic on bovine hemoglobin was stronger than that of 9-hydroxy-dihydroartemisinin. This supports the conclusion that the increase in the number of hydroxyl groups will alleviate the conformation alteration of OVA.

### 3.2. Infrared Spectroscopy and SDS−PAGE Analysis

**Figure 3:**
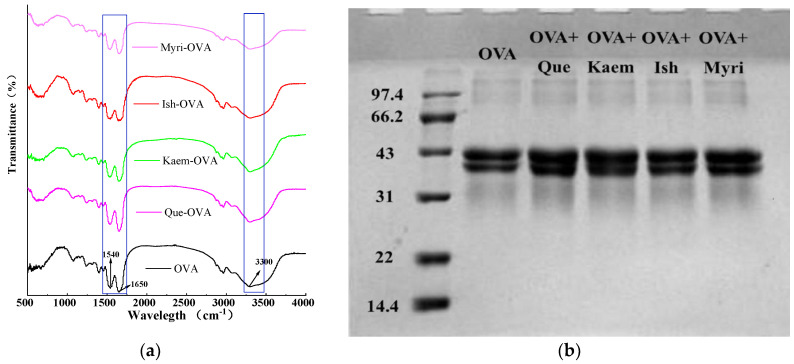
Infrared spectra (**a**) and SDS−PAGE (**b**) of native OVA and OVA−flavonols complexes.

To determine the combination mode of OVA with Que/Ish/Kaem/Myri, the FT−IR spectra and SDS−PAGE of natural OVA and OVA−flavonols complexes were determined. As shown in Figure 3a, the FT−IR spectra of natural OVA and OVA−flavonols both had two strong peaks at ~1650 cm^−1^ and ~1540 cm^−1^, which were the characteristic peaks of amide I (absorption peak of C=O stretching vibration) and amide II (absorption peaks of C−N stretching and N−H bending) [24]. The peak of amide II of N−OVA exhibited an obvious blue shift; the values were shifted from 1540.85 to 1536.99, 1538.92, 1535.1, and 1538.92 cm^−1^, respectively, upon binding with Que/Ish/Kaem/Myri. The absorption peak of O-H stretching at 3300 cm^−1^ also experienced blue shift, and exhibited a similar change trend with that of amide II. Kaem has the greatest influence, and Ish possessed the minimal effect; this is similar with the results of endogenous fluorescence shown in Figure 1. However, no new peak occurred or was weakened obviously after the addition of flavonols, suggesting no consumption or addition of characteristic functional group on OVA. Therefore, it can be inferred that flavonols only altered the spatial structure of OVA, and the combination of OVA with flavonols is noncovalent [14]. Therefore, SDS−PAGE was applied to ensure this speculation and to study whether Que/Ish/Kaem/Myri causes the aggregation or depolymerization of OVA. As shown in Figure 3b, all samples had an obvious protein band at about 45 kDa, the addition of Que/Ish/Kaem/Myri did not cause significant shift on the band, and no new band at nearby 90 kDa was observed. Similar results were found on the interactions of tea polyphenols with ovalbumin and lysozyme [15]. These indicated that Que, Ish, Kaem, and Myri did not induce the aggregation or disaggregation of OVA; the insignificant increase in the molecular weight of OVA further confirmed the bind mode as noncovalent.

### 3.3. CD Spectra Analysis

Effects of Que/Ish/Kaem/Myri on the secondary structure of OVA are shown in Table 1. In terms of N-OVA, the proportion of α-helix, β-sheet, turns, and unordered was about 27.4%, 18.25%, 23.33%, and 31.02%, respectively. This is similar to the results of natural OVA reported by other research [25,26]. After the combination with flavonols, the secondary structure change mainly occurred in the α-helix and β-sheet regions. When Que, Kaem, Ish, and Myri were added, the α-helix content was reduced by 1.56%, 2.64%, 1.28%, and 1.48%, respectively. The β-sheet content was individually increased by 2.22%, 3.04%, 1.41%, and 2.38%. Meanwhile, the content of turn and random coils were almost unchanged. Therefore, Que/Ish/Kaem/Myri could cause the conversion of α-helix to β-sheet. This could be due to the noncovalent binding of Que/Ish/Kaem/Myri with OVA breaking the hydrogen bond in α-helix structure [27], resulting in the loosening of OVA structure [28]. Kaem altered the structure of OVA mostly; the hydroxylation of C-3′ and methoxylation of C-5′ had a weakening effect on the secondary structure changes in OVA, and no significant difference was observed on the secondary structures of OVA in the presence of Que, Myri, and Ish (*p* > 0.05).

### 3.4. Interaction Mechanism of OVA with Que/Kaem/Ish/Myri

#### 3.4.1. Fluorescence Quenching Mechanism

**Figure 4:**
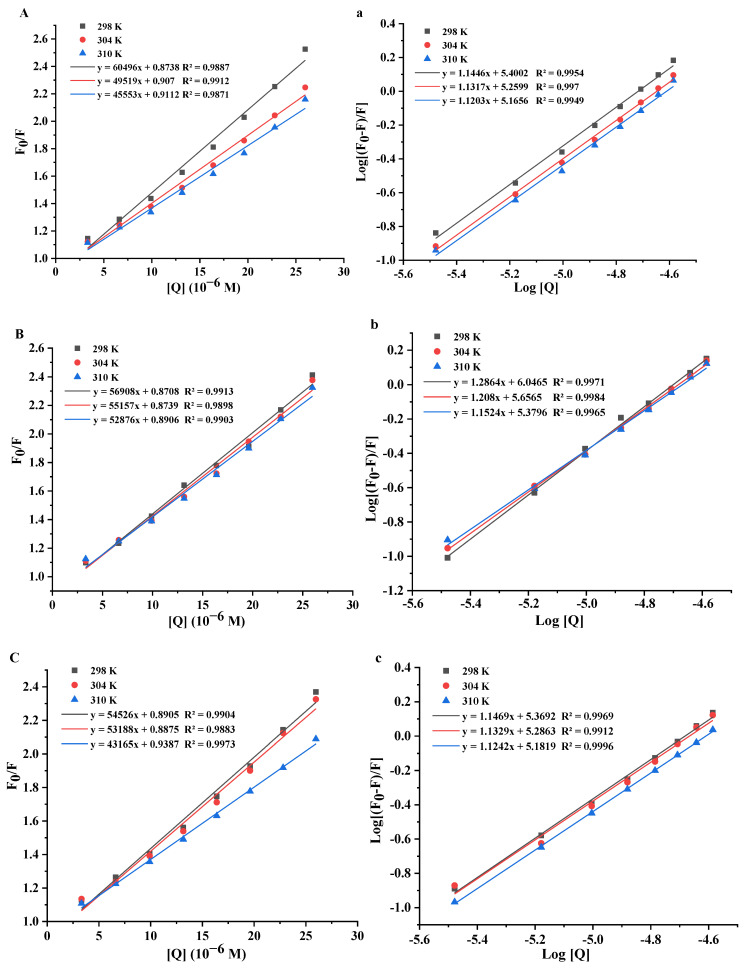

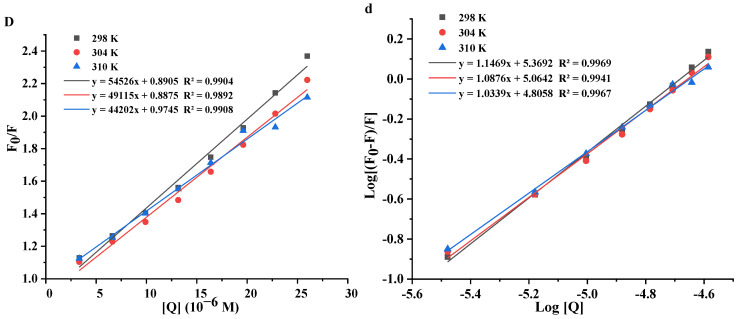
The Stern−Volmer and double logarithmic plots of Que (**A**,**a**), Kaem (**B**,**b**), Ish (**C**,**c**) and Myri (**D**,**d**) quenching the fluorescence of OVA at 298 K, 304 K, and 310 K.

The Trp, Tyr, and Phe are the main luminescent groups in protein, they can be used as an internal fluorescent probe of proteins to detect the interaction information with small molecules, such as fluorescence quenching type, binding constant (*n*), and thermodynamic parameters [29]. The fluorescence quenching type is divided into static and dynamic quenching according to the change trend of fluorescence quenching constant (*K_sv_*) with increasing reaction temperatures and the value of *K_q_*, decreasing *K_sv_*, and high *K_q_* (>2.0 × 10^10^ L/mol/s) reflecting a static quenching [14,16,30]. To demonstrate the fluorescence quenching mechanism of OVA by Que/Ish/Kaem/Myri, the Stern−Volmer graphs at different temperatures (298 K, 304 K, 310 K) were plotted (Figure 4), which showed a good linear relationship, indicating a single endogenous fluorescence quenching way of Que/Ish/Kaem/Myri on OVA [30]. Meanwhile, it can be seen from Table 2 that the *K_sv_* of Que, Kaem, Ish, and Myri reduced gradually with the rising reaction temperatures, and the *K_q_* values ranged from 4.42 to 60.50 × 10^12^ L/moL/s, which were much larger than the maximum collision quenching constant 2.0 × 10^10^ L/mol/s. These illustrated a static quench mechanism of Kaem, Que, Ish, and Myri on OVA fluorescence.

#### 3.4.2. Binding Constants and Binding Sites

The plots of [*Q*] vs. *F*_0_/*F* (Figure 4) and Equation (2) were applied to calculate the quenching constants *K_a_* and the number of binding sites *n* of Que/Ish/Kaem/Myri with OVA at three temperatures; results are listed in Table 2. The *n* of Que/Ish/Kaem/Myri were all detected to be about 1, indicating that Que/Ish/Kaem/Myri bound with OVA by an equal mole manner, that is, one molecule of OVA only binds to one molecule of flavonol [31]. The binding constants *K_a_* of Que/Ish/Kaem/Myri with OVA decreased with the increase in reaction temperatures, indicating that the binding ability of these three flavonols with OVA decreased with the rise in temperatures [15]. In addition, the *K_a_* of Que, Kaem, Ish, and Myri at 304 K was 18.19, 45.34, 19.33, and 11.59 × 10^4^ L/mol, respectively. Therefore, Kaem had the strongest binding ability with OVA, followed by Que, and Myri presented the weakest binding ability. The increase in hydroxyl group and methoxylation on the B-ring leads to the decrease in binding ability; similar results were also found on the binding of flavonoids with other proteins [32]. The addition of hydroxyl groups reduced the hydrophilicity of flavonols, which might weaken the affinity of flavonols with the hydrophobic pocket of OVA. A positive correlation between the binding ability and antioxidant activity of complexes was found in this research. Meanwhile, a contrary result was reported by Stojadinovic, [16], this could be due to the difference in protein structure and micromolecules involved.

#### 3.4.3. Thermodynamic Parameters

The thermodynamic parameters enthalpy (*ΔH°*), free energy (*ΔG°*), and entropy (*ΔS°*) between the interaction of protein and quencher at different temperatures can be used to determine the main driving forces involved in the formation of protein-quencher complexes, which can be calculated through Van ’t Hoff equation. When *ΔH°* > 0 and *ΔS°* > 0, hydrophobic interaction plays a major role; *ΔH°* < 0 and *ΔS°* > 0, electrostatic force is dominant; and *ΔH°* < 0 and *ΔS°* < 0, hydrogen bonding and van der Waals forces contribute greatly to the interaction [33].

The thermodynamic parameters between Que/Ish/Kaem/Myri and OVA are shown in Table 2. The *ΔH°* and *ΔS°* for Que/Ish/Kaem/Myri interacting with OVA were both negative, so Que, Ish, Myri, and Kaem bound with OVA mainly through hydrogen bond and van der Waals force. The *ΔG°* were all negative and increased with the increase in temperature, indicating that the combination of Que/Ish/Myri/kaem with OVA was a spontaneous and exothermic process, which means the stability of Que/Ish/Myri/ Kaem−OVA complexes decreased with the increase in binding temperatures [34], which is consistent with the results of binding constants.

**Table 2:** Binding and thermodynamics parameters between the interaction of OVA with Que, Myri, Kaem, and Ish at different temperatures.

	*T* (K)	*K_sv_* (10^4^ L/moL)	*K_q_* (10^12^ L/moL/s)	*n*	*K_a_* (10^4^ L/moL)	*ΔH°* (kJ/mol)	*ΔS°* (kJ/mol/K)	*R*	*ΔG°* (kJ/mol)
Que	298 K	60.50	60.50	1.145	25.13				−30.77
	304 K	4.95	4.95	1.132	18.19	−34.61	−0.01	0.9898	−30.69
	310 K	4.56	4.56	1.120	14.64				−30.61
Kaem	298 K	5.69	5.69	1.286	111.30				−34.40
	304 K	5.52	5.52	1.208	45.34	−98.38	−0.21	0.9933	−33.11
	310 K	5.29	5.29	1.152	23.75				−31.82
Ish	298 K	5.45	5.45	1.147	23.40				−24.50
	304 K	5.32	5.32	1.133	19.33	−27.58	−0.01	0.994	−24.44
	310 K	4.32	4.32	1.124	15.20				−24.38
Myri	298 K	5.45	5.45	1.147	23.40				−30.60
	304 K	4.91	4.91	1.088	11.59	−83.07	−0.18	0.9987	−29.54
	310 K	4.42	4.42	1.034	6.39				−28.48

#### 3.4.4. Molecular Docking

**Figure 5:**
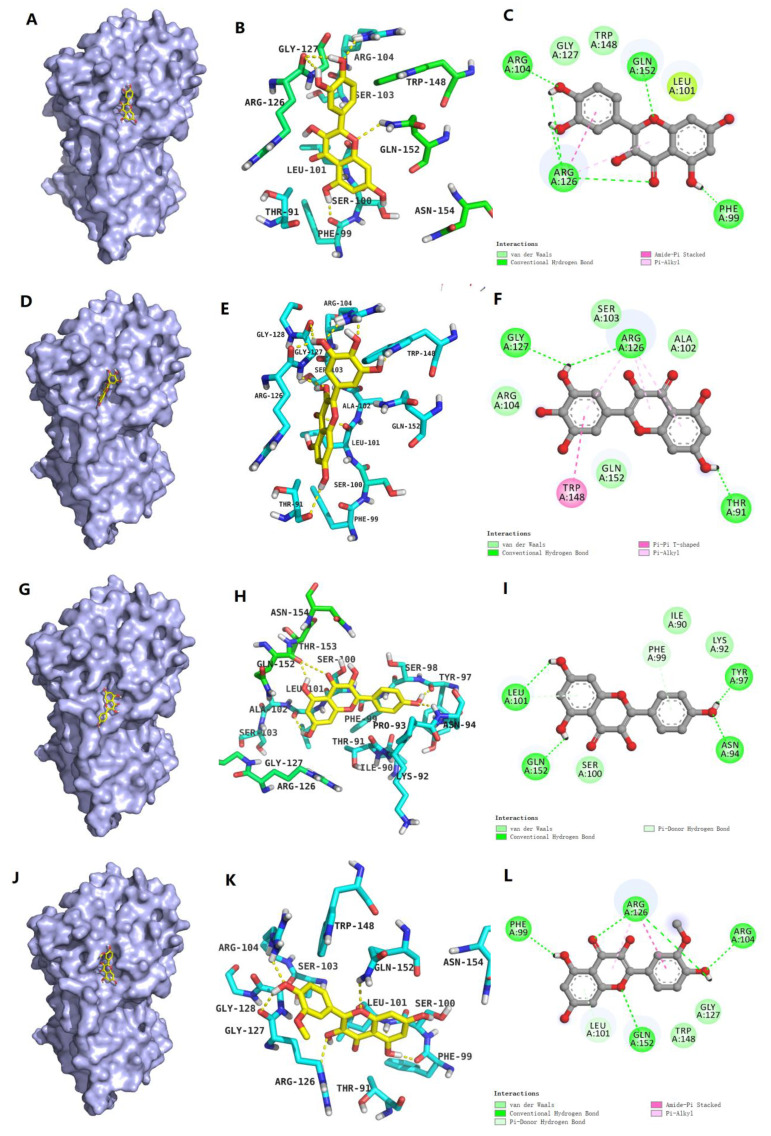
Molecular docking conformation of Que/Ish/Kaem/Myri with OVA. (**A**−**C**): Que−OVA optimal docking conformation; (**D**−**F**): Myri−OVA optimal docking conformation; (**G**−**I**): Kaem−OVA optimal docking conformation; (**J**−**L**): Ish−OVA optimal docking conformation.

In this research, molecular docking was performed to predict the possible interaction sites between Que/Ish/Kaem/Myri and OVA. The optimal docking conformations with the lowest binding energy were visualized by PyMol and are shown in Figure 5. Que, Myri, and Ish bound within the same pocket located on the outer surface of OVA, which consisted of 12 amino acid residues and shared the same amino acid residues of Arg104, Ser103, Trp148, Gln152, Ser100, Phe99, Leu101, Arg126, Gly127, and Thr91. Hydrogen bonding, van der Waals force, and hydrophobic interaction participated in the noncovalent binding of Que/Ish/Myri with OVA. Among them, hydrogen bond and van der Waals force were the main interaction forces, which was consistent with the results of fluorescence titration. While Kaem inserted into the cavity consisted of 17 amino acids, the position was varied from that of Que/Ish/Myri, with seven new amino acid residues Asn94, Lys92, Ile90, Ser98, Tyr97, Pro93, and Thr91 involved in the binding pocket in comparison with Que/Kaem/Myri. This might contribute to the most secondary structure changes of OVA induced by Kaem.

In addition, the formation of hydrogen bonds between Que/Myri/Ish/Kaem and OVA was distinct.Six, three, five, and four hydrogen bonds were formed for Que, Myri, Ish, and Kaem, respectively. The 3′−OH and 7−OH in Myri formed three hydrogen bonds with the Arg126, Gly127, and Thr91 on OVA. The groups in Que and Ish and the amino acid residues in OVA participated in the formation of hydrogen bonds in Que−OVA and Ish−OVA complexes were similar. For Kaem, four hydrogen bonds were formed between the 4′−OH, 5−OH, and 7−OH and Tyr97, Asn94, Gln152, and Leu101. It is clear that only Kaem formed one hydrogen with fluorescent chromophores (Tyr97), which might explain its strongest fluorescent quenching ability on OVA. Unexpectedly, the number of hydrogen bonds formed between Que/Ish/Kaem/Myri and OVA were not positively correlated with their binding constants detected through fluorescence titration assays, nor with the number of hydroxyl groups on the B-ring. This could be explained by the fact that van der Waals force and hydrophobic interactions also contribute to the stability of Que/Ish/Kaem/Myri−OVA complexes. Similar results were observed upon the interaction of apigenin, naringenin, kaempferol, and genistein with β-lactoglobulin [18], as well as the interaction of chrysin, baicalein, and apigenin with purine nucleoside phosphorylase [35].

### 3.5. Antioxidant Activities of Que/Kaem/Ish/Myri−OVA Complexes

**Figure 6:**
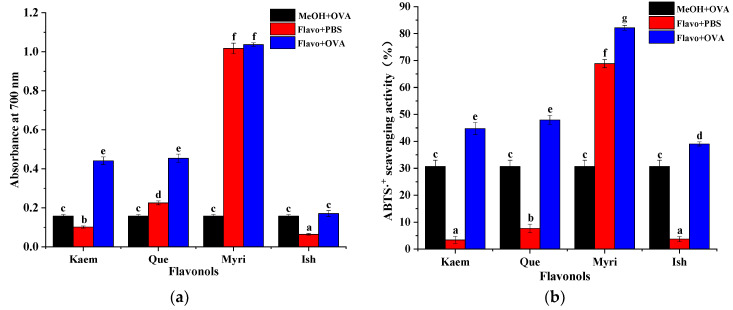
The Fe^3+^ reducing ability (**a**) and ABTS^+^· scavenging activity (**b**) of OVA and OVA complexes. Different lowercase letters (a−g) represent significant differences (*p* < 0.05).

The overproduction of reactive oxygen species is highly related to the pathogenesis of many chronic diseases, such as cancer, diabetes, inflammation, and cardiovascular and neurodegenerative diseases [36,37]; antioxidant activity was, therefore, considered as an important indicator for evaluating the biological activities of natural active compounds or extracts. Antioxidants can reduce Fe^3+^ to blue purple Fe^2+^-TPTZ under acidic conditions. The blue-green ABTS radical cation can be reduced to colorless ABTS under the action of antioxidants. Therefore, Fe^3+^ reducing ability and ABTS^+^· scavenging ability are usually used to detect the antioxidant activity of bioactive constituents. As shown in Figure 6, both OVA and Que/Kaem/Ish/Myri solutions alone showed considerable antioxidant activity. Myri exhibited the strongest activities on the two models, followed by Que, and the weakest ability was found on Ish, suggesting an obvious positive correlation between the antioxidant activity and the number of hydroxyl groups, while methylation of 3′-OH has a significant negative effect on the antioxidant activity.

Hydrogen atom transfer (HAT) and electron transfer (ET) are the major mechanisms of flavonoids to scavenge free radicals. That is, free radicals receive a hydrogen atom or an electron from flavonoids to form stable compound. In Fe^3+^ reduction and ABTS^+^· scavenging experiments, phenolic hydroxyl groups (ArOH) of flavonoids were cleaved to form H^+^ and ArO^−^, Fe^3+^, and ABTS^+^· receive an electron from ArO^−^ to form Fe^2+^ and stable ABTS, while flavonoids form stable and nontoxic ArO· [38]. Therefore, the more phenolic hydroxyl groups there are in flavonoids, the stronger the antioxidant activity will be. Heim et al. [22] found that flavonoids with more hydroxyl groups on the B-ring had higher antioxidant capacity. Methylation of phenolic hydroxyl groups on the B-ring of flavonoids change the redox potential, thus reducing the free radical scavenging ability [39].

Upon the addition of OVA, the reducing ability of Kaem−OVA and Que−OVA complexes were much higher than that of Kaem/Que and OVA solution alone; the ABTS^+^· scavenging ability of flavonols−OVA complexes were all stronger than corresponding Que/Ish/Kaem/Myri and OVA solution, whereas the Myri−OVA complexes showed similar reducing ability with Myri solution. However, the Fe^3+^ reducing and ABTS^+^· scavenging activity of OVA were even higher than those of Kaem and Ish solution, especially for ABTS^+^· scavenging activity. Thus, to evaluate the additive or masking effect of OVA on the antioxidant activity of Que/Kaem/Ish/Myri, the contribution of OVA must be taken into consideration. The increment in total antioxidant capacity (*AC*) was calculated by the following equation:

$$AC\;\left(\%\right)=\frac{AC_{1}-AC_{2}-AC_{3}}{AC_{2}+AC_{3}}$$

where *AC*_1_, *AC*_2_, and *AC*_3_ are the antioxidant capacity of OVA−flavonols, flavonols, and OVA, respectively.

After the addition of Que, Ish, Kaem, and Myri to OVA solution, the Fe^3+^ reducing capacity of the mixtures increased by 18.68%, −22.9%, 70.39%, and −11.75%, respectively, and the ABTS^+^· scavenging capacity increased by 25.08%, 13.56%, 31.3%, and −17.42%, respectively. These mean that the *AC* of Que and Kaem were enhanced after the addition of OVA, and the biggest additive effect was observed on Kaem, while the antioxidant activity of Myri was masked after binding with OVA. Meanwhile, an inconsistent result was detected on the radical scavenging and reducing ability of Ish; the latter was masked. Therefore, OVA−flavonols interaction can alter the antioxidant activity of flavonols, but the degree critically depends on the structure of flavonoids involved [6]. Amorati et al. [40] also reported that intra-molecular or inter-molecular noncovalent interactions can modify the antioxidant activity of polyphenols. The additive effect of Kaem−OVA and Que−OVA complexes may be due to the relative higher binding of Kaem/Que with OVA, which might improve their water solubility, leading to enhanced antioxidant capacity [41]. As a water-soluble flavonoid, antioxidant shielding effect was observed on Myri after interacting with OVA. A similar antioxidant masking effect was found on bovine serum albumin−Myri complexes [42]. In addition, Kaem altered the conformation and secondary structure of OVA most (shown in Figure 2 and Table 1), which might promote the exposure of hydrophilic groups and provide more hydrogen or electron donors [40,43].

## 4. Conclusions

This research firstly investigated the influence of OVA on the antioxidant activity of flavonols with different hydroxyl substitution on the B-ring and the interaction mechanism. Que, Ish, Kaem, and Myri noncovalently bound to OVA through a spontaneous exothermic process. Hydrogen bonding and van der Waals force were the major driving forces, and six, five, four, and three hydrogen bonds were formed individually. The binding abilities of Que/Kaem/Ish/Myri−OVA complexes at 298 K were positively correlated to their antioxidant activity. Obvious antioxidant additive effect was observed on OVA−Que and OVA−Kaem complexes; Kaem presented the most additive effect, and the Fe^3+^ reducing and ABTS^+^· scavenging activity was increased by 70.39% and 25.08%, respectively. However, Myri−OVA interaction indicated an antioxidant masked effect. Que/Ish/Kaem/Myri could alter the conformational structure of OVA and induce the transformation of α-helix to β-sheet; Kaem caused the biggest alteration. The hydroxylation of C−3′ and methoxylation of C−5′ of Kaem would reduce its binding ability with OVA and decrease its influence on the microenvironment and secondary structure of OVA; the antioxidant activity with OVA complexes was also reduced. This may provide a new idea for OVA as a carrier to transport hydrophobic drugs in vivo and for improving the antioxidant activity of flavonols.

## Figures and Tables

**Table 1:** Influence of Que, Kaem, Ish and Myri on the secondary structure of OVA.

Sample	Secondary Structure			
	α-Helix (%)	β-Sheet (%)	Turns (%)	Unordered (%)
OVA	27.40 ± 1.60 ^b^	18.25 ± 2.22 ^a^	23.33 ± 0.37 ^a^	31.02 ± 0.29 ^a^
OVA+Que	25.84 ± 0.90 ^ab^	20.47 ± 1.02 ^ab^	23.38 ± 0.22 ^a^	30.35 ± 0.10 ^a^
OVA+Kaem	24.76 ± 0.31 ^a^	21.29 ± 0.19 ^a^	22.84 ± 0.45 ^a^	31.09 ± 0.60 ^a^
OVA+Ish	26.12 ± 0.79 ^ab^	19.66 ± 1.36 ^ab^	23.61 ± 0.65 ^a^	30.62 ± 0.73 ^a^
OVA+Myri	25.92 ± 0.83 ^ab^	20.63 ± 1.36 ^ab^	22.84 ± 0.23 ^a^	30.61 ± 0.69 ^a^

Note: different lowercase letters (a, b) represent significant differences (*p* < 0.05).

## Data Availability

The data are available from the first author or corresponding authors upon request.

## References

[1] Jiang B., Song J.-L., Jin Y.-C. (2020). A flavonoid monomer tricin in *Gramineous* plants: Metabolism, bio/chemosynthesis, biological properties, and toxicology. Food Chem..

[2] Lin S.-Y., Zhang G.-W., Liao Y.-J., Pan J.-H., Gong D.-M. (2015). Dietary flavonoids as xanthine oxidase inhibitors: Structure-affinity and structure-activity relationships. J. Agric. Food Chem..

[3] Lerman J.S., Haslem J., Kim L., Ponderelli J., Ma M., Felson R., Sanderson K., Murphy K., Hopkins K., Wright E. (2015). Collected research on phytonutrients: Flavonoids. J. Culin. Sci. Technol..

[4] Survay N.S., Upadhyaya C.P., Kumar B., Young K.E., Yoon D.Y., Park S.W. (2011). New genera of flavonols and flavonol derivatives as therapeutic molecules. J. Korean Soc. Appl. Biol..

[5] Dabeek W.M., Marra M.V. (2019). Dietary quercetin and kaempferol: Bioavailability and potential cardiovascular-related bioactivity in humans. Nutrients.

[6] Zhang Q.-Z., Cheng Z.-Z., Wang Y.-B., Fu L.-L. (2020). Dietary protein-phenolic interactions: Characterization, biochemical-physiological consequences, and potential food applications. Crit. Rev. Food Sci..

[7] Teng H., Zheng Y.-M., Cao H., Huang Q., Xiao J.-B., Chen L. (2021). Enhancement of bioavailability and bioactivity of diet-derived flavonoids by application of nanotechnology: A review. Crit. Rev. Food Sci..

[8] Tsuchiya H. (2010). Structure-dependent membrane interaction of flavonoids associated with their bioactivity. Food Chem..

[9] Kovacs-Nolan J., Phillips M., Mine Y. (2005). Advances in the value of eggs and egg components for human health. J. Agr. Food Chem..

[10] Mine Y. (2002). Recent advances in egg protein functionality in the food system. World Poult. Sci. J..

[11] Gou S.-Q., Chen Q.-B., Liu Y., Zeng L., Song H.-L., Xu Z.-G., Kang Y.-J., Li H.-M., Xiao B. (2018). Green fabrication of ovalbumin nanoparticles as natural polyphenol carriers for ulcerative *Colitis Therapy*. ACS Sustain. Chem. Eng..

[12] Liu Y.-J., Ying D.-Y., Cai Y.-X., Le X.-Y. (2017). Improved antioxidant activity and physicochemical properties of curcumin by adding ovalbumin and its structural characterization. Food Hydrocolloid.

[13] Zhang L., Zhou W.-N., Tu Z.-C., Yang S.-H., Xu L., Yuan T. (2020). Influence of hydroxyl substitution on the suppression of flavonol in harmful glycation product formation and the inhibition mechanism revealed by spectroscopy and mass spectrometry. J. Agric. Food Chem..

[14] Zhang L., Lu Y., Ye Y.-H., Yang S.-H., Tu Z.-C., Chen J., Wang H., Wang H.-H., Yuan T. (2019). Insights into the mechanism of quercetin against BSA-fructose glycation by spectroscopy and high-resolution mass spectrometry: Effect on physicochemical properties. J. Agric. Food Chem..

[15] Shen F., Niu F.-G., Li J.-H., Su Y.-J., Liu Y.-T., Yang Y.-J. (2014). Interactions between tea polyphenol and two kinds of typical egg white proteins—ovalbumin and lysozyme: Effect on the gastrointestinal digestion of both proteins in vitro. Food Res. Int..

[16] Stojadinovic M., Radosavljevic J., Ognjenovic J., Vesic J., Prodic I., Stanic-Vucinic D. (2013). Binding affinity between dietary polyphenols and β-lactoglobulin negatively correlates with the protein susceptibility to digestion and total antioxidant activity of complexes formed. Food Chem..

[17] Liu Y.-J., Cai Y.-X., Ying D.-Y., Fu Y.-L., Xiong Y.-H., Le X.-Y. (2018). Ovalbumin as a carrier to significantly enhance the aqueous solubility and photostability of curcumin: Interaction and binding mechanism study. Int. J. Biol. Macromol..

[18] Li T., Hu P., Dai T.-T., Li P.-Y., Ye X.-Q., Chen J., Liu C.-M. (2018). Comparing the binding interaction between *β*-lactoglobulin and flavonoids with different structure by multi-spectroscopy analysis and molecular docking. Spectrochim. Acta A.

[19] Zhang L., Tu Z.-C., Xie X., Lu Y., Wang Z.-X., Wang H., Sha X.-M. (2016). Antihyperglycemic, antioxidant activities of two *Acer palmatum* cultivars, and identification of phenolics profile by UPLC-QTOF-MS/MS: New natural sources of functional constituents. Ind. Crop. Prod..

[20] Zhang L., Tu Z.-C., Wang H., Kou Y., Wen Q.-H., Fu Z.-F., Chang H.-X. (2015). Response surface optimization and physicochemical properties of polysaccharides from *Nelumbo nucifera* leaves. Int. J. Biol. Macromol..

[21] Huang X.-Q., Tu Z.-C., Xiao H., Wang H., Zhang L., Hu Y.-M., Zhang Q.-T., Niu P.-P. (2012). Characteristics and antioxidant activities of ovalbumin glycated with different saccharides under heat moisture treatment. Food Res. Int..

[22] Wang R.-Q., Yin Y.-J., Li H., Wang Y., Pu J.-J., Wang R., Dou H.-J., Song C.-J., Wang R.-Y. (2013). Comparative study of the interactions between ovalbumin and three alkaloids by spectrofluorimetry. Mol. Biol. Rep..

[23] Xiao M.-S., Han L.-N., Zhou L., Zhou Y.-H., Huang X.-Q., Ge X.-F., Wei S.-H., Zhou J.-H., Wu H.-M., Shen J. (2014). Comparison and investigation of bovine hemoglobin binding to dihydroartemisinin and 9-hydroxy-dihydroartemisinin: Spectroscopic characterization. Spectrochim. Acta A.

[24] Tang S.-T., Yu J.-L., Lu L.-Z., Fu X., Cai Z.-X. (2019). Interfacial and enhanced emulsifying behavior of phosphorylated ovalbumin. Int. J. Biol. Macromol..

[25] Xiong W.-F., Wang Y.-T., Zhang C.-L., Wan J.-W., Shah B.R., Pei Y.-Q., Zhou B., Li J., Li B. (2016). High intensity ultrasound modified ovalbumin: Structure, interface and gelation properties. Ultrason. Sonochem..

[26] Huang Q., Ma M.-H., Cai Z.-X., Luo Z., Huang X., Sun S.-G. (2011). Effect of s-configuration transformation on the microstructure of ovalbumin. Spectrosc. Spect. Anal..

[27] Sheng L., Tang G.-Y., Wang Q., Zou J., Ma M.-H., Huang X. (2020). Molecular characteristics and foaming properties of ovalbumin-pullulan conjugates through the *Maillard* reaction. Food Hydrocolloid.

[28] Bi H.-N., Tang L., Gao X., Jia J.-J., Lv H.-H. (2016). Spectroscopic analysis on the binding interaction between tetracycline hydrochloride and bovine proteins β-casein, α-lactalbumin. J. Lumin..

[29] Chen T.-T., Zhu S.-J., Cao H., Shang Y.-F., Wang M.-A., Jiang G.-Q., Shi Y.-J., Lu T.-H. (2011). Studies on the interaction of salvianolic acid B with human hemoglobin by multi-spectroscopic techniques. Spectrochim. Acta A.

[30] Ding F., Peng W., Peng Y.-K. (2016). Biophysical exploration of protein-flavonol recognition: Effects of molecular properties and conformational flexibility. Phys. Chem. Chem. Phys..

[31] Lu Y., Wang Y.-L., Gao S.-H., Wang G.-K., Yan C.-L., Chen D.-J. (2009). Interaction of quercetin with ovalbumin: Spectroscopic and molecular modeling studies. J. Lumin..

[32] Wu S.-M., Zhang Y.-Y., Ren F.-Z., Qin Y.-H., Liu J.-X., Liu J.-W., Wang Q.-Y., Zhang H. (2018). Structure-affinity relationship of the interaction between phenolic acids and their derivatives and β-lactoglobulin and effect on antioxidant activity. Food Chem..

[33] Wang Q., Liu X.-Y., Su M., Shi Z.-H., Sun H.-W. (2015). Study on the interaction characteristics of cefamandole with bovine serum albumin by spectroscopic technique. Spectrochim. Acta A.

[34] Yu X., Cai X., Li S., Luo L.-Y., Wang J., Wang M., Zeng L. (2021). Studies on the interactions of theaflavin-3,3′-digallate with bovine serum albumin: Multi- spectroscopic analysis and molecular docking. Food Chem..

[35] Wen Q.H., Wang L.H., Zeng X.A., Niu D.B., Wang M.S. (2018). Hydroxyl-related differences for three dietary flavonoids as inhibitors of human purine nucleoside phosphorylase. Int. J. Biol. Macromol..

[36] Liu J.-G., Li Y.-J., Chen S., Lin Y.-P., Lai H.-Q., Chen B.-L., Chen T.-F. (2020). Biomedical application of reactive oxygen species-responsive nanocarriers in cancer, inflammation, and neurodegenerative diseases. Front. Chem..

[37] Pisoschi A.M., Pop A., Iordache F., Stanca L., Predoi G., Serban A.I. (2021). Oxidative stress mitigation by antioxidants—An overview on their chemistry and influences on health status. Eur. J. Med. Chem..

[38] Vaganek A., Rimarcik J., Lukes V., Klein E. (2012). On the energetics of homolytic and heterolytic O-H bond cleavage in flavonoids. Comput. Theor. Chem..

[39] Seeram N.P., Nair M.G. (2002). Inhibition of lipid peroxidation and structure-activity-related studies of the dietary constituents anthocyanins, anthocyanidins, and catechins. J. Agric. Food Chem..

[40] Amorati R., Valgimigli L. (2012). Modulation of the antioxidant activity of phenols by non-covalent interactions. Org. Biomol. Chem..

[41] Qie X.J., Chen W.P., Zeng M.M., Wang Z.J., Chen J., Goff H.D., He Z.Y. (2021). Interaction between beta-lactoglobulin and chlorogenic acid and its effect on antioxidant activity and thermal stability. Food Hydrocolloid.

[42] Geng R., Ma L., Liu L.L., Xie Y.X. (2019). Influence of bovine serum albumin-flavonoid interaction on the antioxidant activity of dietary flavonoids: New evidence from electrochemical quantification. Molecules.

[43] Liu J., Tu Z., Shan Y.-H., Wang H., Liu G.-X., Sha X.-M., Zhang L., Yang P. (2017). Improved antioxidant activity and glycation of alpha-lactalbumin after ultrasonic pretreatment revealed by high-resolution mass spectrometry. J. Agric. Food Chem..

